# *Onchocerca lupi* Nematode in Cat, Portugal

**DOI:** 10.3201/eid2112.150061

**Published:** 2015-12

**Authors:** Carla Maia, Giada Annoscia, Maria Stefania Latrofa, André Pereira, Alessio Giannelli, Laurentina Pedroso, Domenico Otranto

**Affiliations:** Universidade Nova de Lisboa, Lisbon, Portugal (C. Maia);; Universidade Lusófona de Humanidades e Tecnologias, Lisbon (C. Maia, A. Pereira, L. Pedroso);; Università degli Studi di Bari, Valenzano, Italy (G. Annoscia, M.S. Latrofa, A. Giannelli, D. Otranto)

**Keywords:** cats, Onchocerca lupi, nematode, Portugal, zoonoses, parasites, felids

**To the Editor:** *Onchocerca lupi* (Spirurida, Onchocercidae) is a nematode that infects the ocular tissues of dogs and humans. This filarioid remained almost unknown until recently, when it was reported in dogs from Europe and North America (*1**–**3*). *O. lupi* was also detected in 2 cats from the United States (*4*), which suggests that not only canids but also felids are suitable hosts for this little-known nematode. In addition, the zoonotic potential of *O. lupi* nematodes was demonstrated in human patients from Iran, Tunisia, Turkey, and the United States (*3**,**5*).

Clinical signs of canine onchocercosis include conjunctivitis, exophthalmos, periorbital swelling, photophobia, discomfort, lacrimation, ocular discharge, subconjunctival granuloma, ulcerative keratitis, and anterior and posterior uveitis (*1*). Signs in cats are similar to those in dogs (*4*).

After the first case of canine ocular onchocercosis was reported in the Algarve region in southern Portugal (*6*), a survey to detect microfilariae in apparently healthy dogs revealed an 8.3% prevalence of infection (*7*). Because no data regarding *O. lupi* nematode infection in cats from Europe are available, the aim of this study was to evaluate the infection’s occurrence in cats in Portugal, where canine infection has been previously reported (*8*).

In October 2014, a total of 155 stray cats were sampled from Praia de Faro in the Algarve (37°0′29.4546′′N, 7°59′41.265′′W, altitude 9 meters). The sampling area is a small peninsula within an area characterized by a line of sand dunes formed by peninsulas and sandy islands that protect a vast area of marshland, canals, and islets from the Atlantic Ocean. All stray cats were captured under the scope of a trap, neuter, and return project. This study was approved by the ethical committee of the Faculty of Veterinary Medicine, Universidade Lusófona de Humanidades e Tecnologias.

Ear tipping is commonly done in trap, neuter, and return programs to identify cats that have been sterilized. These skin samples (0.5 cm^2^) were soaked at room temperature in 1 mL of saline solution, and sediments were individually observed under light microscopy (*9*).

Of 155 cats, 1 (0.65%) with no clinical signs of ocular infection was positive for *O. lupi* microfilariae. Microfilariae were identified according to morphologic keys (*9*) and differentiated from those of other filarioid species infecting cats in the Mediterranean region. *O. lupi* microfilariae had a short, flattened, unsheathed body (mean length 110.1 ± 7.5 μm, width 6.8 ± 1.2 μm) with a rounded head bearing a tiny tooth on the cephalic edge. The body was blunt with a short bent tail of ≈11.7 μm.

After we made microscopic observations, skin samples were processed as described elsewhere (*10*). Partial cytochrome *c* oxidase subunit 1 (*cox*1) gene fragments (689 bp) were amplified (*10*). In accordance with the morphologic identification, BLAST analysis (http://blast.ncbi.nlm.nih.gov/Blast.cgi) of *cox*1 gene showed a high overall nucleotide homology with sequences of *O. lupi* available in GenBank. All *cox*1 sequences available in GenBank for *O. lupi* nematodes were analyzed by using MEGA6 (http://www.megasoftware.net) and showed a low intraspecific variability, ranging from 0% to 2.1% (mean 0.7%). Phylogenetic analysis of *cox*1 sequences with MEGA6 and the neighbor-joining method confirmed that the sequence obtained clustered with that of *O. lupi* nematodes from Portugal available in GenBank (Figure). The obtained sequence was deposited in GenBank (accession no. KP453715).

**Figure:**
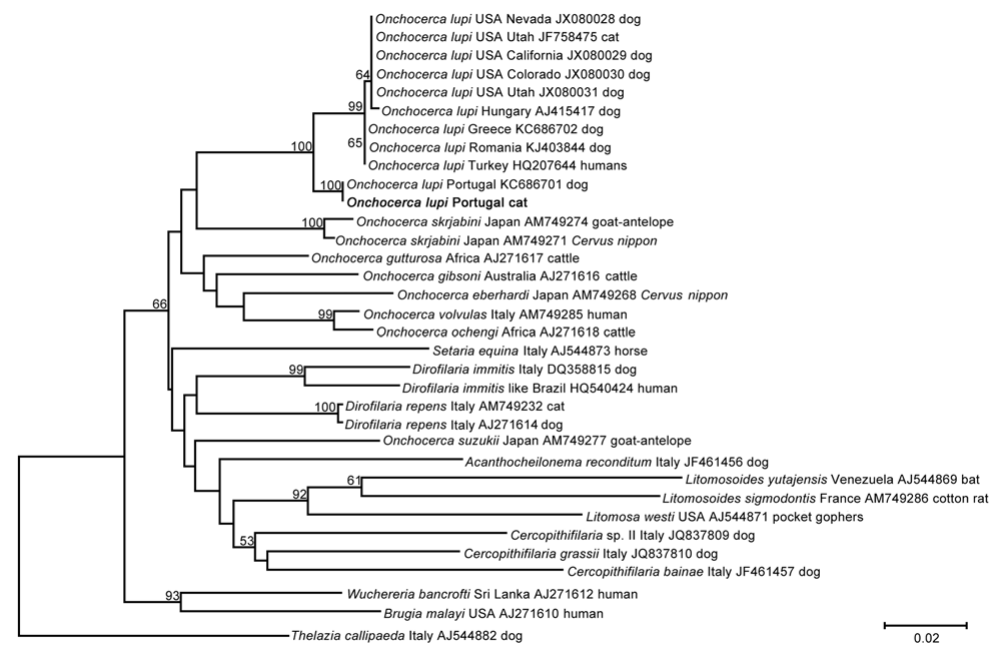
Phylogenetic analysis of partial cytochrome *c* oxidase subunit 1 gene segment (689 bp) of *Onchocerca lupi* isolated from a cat in Portugal (bold) compared with segments from other nematodes and roundworms retrieved from GenBank (accession numbers indicated). Numbers along branches are bootstrap values. Scale bar indicates nucleotide substitutions per site.

We describe detection of *O. lupi* nematodes in a cat from Europe. The complete life cycle of *O. lupi* nematodes remains unknown, although arthropods should act as a vector (*2**,**4**,**7*). Because most of the potential vectors (i.e., black flies, mosquitoes, and biting midges) increase their activity during spring and summer, we cannot rule out that skin sampling conducted in late October affected the chance to detect additional infected animals. In addition, sampling was performed during the day, instead of late afternoon or night, when the number of microfilariae is higher (*7*), which might account for the low prevalence of infection obtained in this study.

As previously reported for most infected dogs from the same area, the infected cat lacked apparent clinical signs of infection, suggesting that subclinically infected animals might be carriers and reservoirs of *O. lupi* nematodes (*7*). Further investigation such as population-based surveys should be performed to estimate the distribution of the infection in cats and dogs and to assess the risk to humans.

Detection of *O. lupi* nematodes in dogs and cats from Algarve confirms that this parasite is endemic to southern Portugal. Veterinarians, local pet owners, and tourists (particularly those from countries where the disease is not endemic and who bring their pets) should be alerted to the risk for infection by this filarioid and the need to implement measures to protect animals and persons. Physicians and ophthalmologists should include this zoonosis in the differential diagnosis for ocular nodular lesions, particularly in patients from areas where *O. lupi* nematodes have been reported.
